# Neuraxial opioids as analgesia in labour, caesarean section and hysterectomy: A questionnaire survey in Sweden

**DOI:** 10.12688/f1000research.10705.2

**Published:** 2017-03-28

**Authors:** Anette Hein, Caroline Gillis-Haegerstrand, Jan G. Jakobsson

**Affiliations:** 1 Department of Anaesthesia & Intensive Care, Danderyds Hospital, Stockholm, 182 88, Sweden

**Keywords:** intrathecal morphine, labour pain, postoperative pain, Caesarean Section, hysterectomy, sufentanil, fentanyl, epidural morphine

## Abstract

*Background*: Neuraxial opioids improve labour analgesia and analgesia after caesarean section (CS) and hysterectomy. Undesirable side effects and difficulties in arranging postoperative monitoring might influence the use of these opioids. The aim of the present survey was to assess the use of intrathecal and epidural morphine in gynaecology and obstetrics in Sweden. *Methods*: A questionnaire was sent to all anaesthetic obstetric units in Sweden concerning the use and postoperative monitoring of morphine, sufentanil and fentanyl in spinal/epidural anaesthesia. *Results*: A total of 32 of 47 (68%) units responded representing 83% of annual CS in Sweden. In CS spinal anaesthesia, 20/32 units use intrathecal morphine, the most common dose of which was 100 μg (17/21). Intrathecal fentanyl (10-20 μg) was used by 21 units and sufentanil (2.5 -10 μg) by 9/32 of the responding units. In CS epidural anaesthesia, epidural fentanyl (50-100 μg) or sufentanil (5-25 μg) were commonly used (25/32), and 12/32 clinics used epidural morphine, the majority of units used a 2 mg dose. Intrathecal morphine for hysterectomy was used by 20/30 units, with 200 μg as the most common dose (9/32). Postoperative monitoring was organized in adherence to the National Guidelines; the patient is monitored postoperative care or an obstetrical ward over 2-6 hours and up-to 12 hours in an ordinary surgical ward. Risk of respiratory depression/difficult to monitor was a reason for not using intrathecal opioids. *Conclusions*: Neuraxial morphine is used widely in Sweden in CS and hysterectomy, but is still restricted in some units because of the concern for respiratory depression and difficulties in monitoring.

## Introduction

Intrathecal and epidural morphine improve postoperative analgesia after caesarean section (CS) and hysterectomy and during intrathecal labour analgesia ^1– 3^. In 1981, the Swedish Society of Anaesthetists conducted a nationwide survey of experience with intrathecal and extradural opiates ^4^. They found intrathecal morphine was administered in total to only 90–150 patients in 10 of 93 responding units, and ventilatory depression requiring treatment with naloxone was needed in six of these patients. Since then, the use of intrathecal and epidural morphine has expanded, with a decrease in the doses used ^5^. Still, the use of intrathecal and epidural morphine in these patient categories vary and the general use in Sweden at present is unknown. Spinal morphine may have undesirable side effects, such as nausea and vomiting, with the most feared side effect being respiratory depression, which is why extended postoperative monitoring is required ^6^. Extended postoperative monitoring demands personal resources and is sometimes difficult to arrange, which may influence the use of spinal morphine.

The primary aim of the present questionnaire survey was to assess the use of intrathecal and epidural morphine in obstetric and gynaecological patients, factors that limit/holdback its use and monitoring routines implemented. Additional aim was to assess the use of other opioids with more rapid onset and shorter duration for these patients.

## Methods

This study was conducted in accordance with the principles outlined in the Declaration of Helsinki. Ethical committee approval was not applied for the present study, since the survey concerns only clinical practice and routines and not patient data in accordance to Swedish ethical board guidelines. The permission to carry out the research was obtained from the head of department of each hospital.

### Survey participants

A questionnaire survey was sent to anaesthesiologists in charge of obstetric anaesthesia units in hospitals in Sweden. In all, 47 obstetric units were identified by the Swedish Medical Birth Register from National Board of Health and Welfare (Sweden).

We identified the anaesthesiologist in charge of the obstetric and gynaecological anaesthesia for each unit by address lists used by Swedish Society of Obstetric Anaesthesia and Intensive Care ( SFOAI), and if these were not present the hospital was phoned to get hold of the anaesthesiologist in charge.

### Survey questionnaire

The survey consisted of 26 questions sent by email to the anaesthesiologists identified in December 2014, and this was repeated in April 2015 to those clinics that had not answered the first questionnaire. The second time the same questionnaire was sent by email and by postal mail, including a return envelope. The email included a letter to the anaesthesiologist and the survey in two versions. The first version was a Word document, possible to fill in by the computer, save and return by email to a special email address for the study purpose only. The second version was a PDF-file that could be printed and filled in by hand and send by post to our hospital address.

The questionnaire ( Supplementary File 1 and Supplementary File 2) was designed to reflect how common the use of intrathecal and epidural morphine, fentanyl and sufentanil as adjunct to local anaesthetics for perioperative care of CS and hysterectomy is, and included questions about the routine use of intrathecal morphine for labour analgesia. The anaesthesiologists were asked to approximate the numbers of CS and hysterectomies performed in spinal and epidural anaesthesia, respectively, and specify the numbers of patients administered with opioids, including neuraxial morphine, for the operations performed. We also asked for doses when spinal/epidural opioids are used. When spinal/epidural morphine is marked “not used” we asked for the reasons behind withholding it. Both multiple choice and written answers were collected. Questions about the organisation of monitoring after neuraxial morphine administration, and known serious adverse events that had occurred in their units were also included.

We calculated the size of the different units by using annual total number of performed CS in the units, in order to put our findings, with regards to the routine use of opioids, into perspective. The annual numbers are collected from The Swedish Medical Birth Register from National Board of Health and Welfare (Sweden).

### Statistical analysis

Data is presented as number and percentage, and range as applicable. No formal statistical tests were used.

## Results

### Responding clinics

In total, 32 units of 47 responded to the questionnaires (68%) representing 83% of annual CS in Sweden. Units with a large number of births and CS were more willing to respond. Among the 21 units with more than 2000 births/year 19 units responded (90%) versus 12/25 (48%) units with less than 2000 births/year. In units performing more CS than 300/year 23/28 units responded (82%) versus 9/19 (47%) units with less than 300/year. In the 15 units performing more than 400 CS/year we got 100% response.

### Caesarean sections (CS)

The routine use of intrathecal opioids in CS is shown in Table 1. All responding units use at least one opioid as adjunct to local anaesthesia in CS.

**Table 1.** Neuroaxial opioids in Ceasarean section. IT = intrathecal, EDA = epidural, M= morphine; F= fentanyl; S= sufentanil.

Neuroaxial opioids in Ceasarean section			Units N
Responding rate			32/47
Intrathecal	Morphine	IT morphine	20
		IT morphine dose 100 µg 125 µg	17 3
		IT morphine but no IT fentanyl/sufentanil	2
	M+F	IT morphine + fentanyl	16
	M+S	IT morphine + sufentanil	2
	Fentanyl	IT fentanyl	21
		IT fentanyl dose 10–14 µg 15–20 µg	18 3
		IT fentanyl but no IT morphine	5
	Sufentanil	IT sufentanil	9
		IT sufentanil dose 2.5 µg 5 µg 5–10 µg	4 4 1
		IT sufentanil but no IT morphine	7
Epidural	M	EDA morphine dose 1 mg 2 mg 4 mg	1 10 1
	F	EDA fentanyl 50–100 µg	10
	S	EDA sufentanil dose 5–10 µg 15–25 µg 25–50 µg	11 3 1

***Intrathecal morphine.*** A total of 20 out of 32 units reported the use of intrathecal morphine as routine in CS spinal analgesia. Three units that did not use intrathecal morphine for CS patients commented that they intended to start using intrathecal morphine within the next year.

For CS the most common intrathecal morphine dose was 100 μg, which was used in 17/20 units and three units used 125 μg. All, except for two units, used morphine in combination with either fentanyl or sufentanil. One of these units reported that they plan to start the addition of fentanyl.

***Intrathecal fentanyl.*** Addition of fentanyl in intrathecal CS anaesthesia was used in 21/32 units; 5 units used fentanyl, but no morphine. In total, 18 units used 10–12.5 microgram dose and 3 units used a 15–20 µg dose.

***Intrathecal sufentanil.*** The addition of sufentanil in intrathecal CS anaesthesia was less commonly used; 9/32 units, where seven units use sufentanil as the sole opioid added. Four units used a 2.5 µg dose, 4 units used a 5 µg dose and one unit used a 5–10 µg dose of sufentanil.

Thus, 16 units used the combination morphine/fentanyl, 2 units used the combination morphine/sufentanil and 2 solely used morphine, 5 solely used fentanyl and 7 solely used sufentanil.

***Epidural morphine.*** Addition of epidural morphine to CS performed in epidural anaesthesia was less common than intrathecal morphine in spinal anaesthesia: 12/32 units added morphine to the epidural local anaesthesia. The majority, 10 units, administered 2 mg dose of morphine, one unit used 1 mg and one unit used a 4 mg dose of morphine.

***Epidural fentanyl.*** Ten units added fentanyl (50–100 µg) in the epidural for CS anaesthesia.

***Epidural sufentanil.*** In total, 15 units used sufentanil as the opioid with rapid onset of action in the epidural anaesthesia for CS anaesthesia, where 11 used doses up to 10 µg, 3 units administered doses up to 25 µg, and one unit used a 25–50 µg dose as the sole opioid added.

***Labour analgesia.*** None of the units used morphine in spinal labour analgesia.

### Hysterectomy

***Intrathecal morphine.*** Spinal anaesthesia with morphine added is used in 20 of the 32 answering units in hysterectomy. The most common dose was 200 µg administered by 9 clinics, 5 clinics used 120–140 µg, 5 used 100 µg and one unit administered 80 µg.

In other types of gynaecological operations, like perineoraphies, malign robot surgery, all gynaecological abdominal surgery 7/32 clinics used intrathecal morphine. One of 32 clinics used epidural morphine, for malign gynaecological surgery.

***Postoperative monitoring.*** Postoperative monitoring was generally organised within the initial 2–6 hours in the postoperative ward and the following hours, up to 12 hours, in the regular surgical ward, according to the guidelines of – Swedish Society of Anaesthesia and Intensive Care ( SFAI). In case of CS the initial 2–6 hours of postoperative monitoring were located to either the obstetrical ward (as it was in eight hospitals as routine and in some hospitals occasionally) or the postoperative ward and the following hours, up to 12 hours, in the regular ward.

Seven of the 12 units that did not use spinal/epidural morphine describe risk of respiratory depression and difficulties to monitor as the main reasons for withholding its use. One unit answered they routinely used intrathecal morphine for CS but did not present any dose of intrathecal morphine, only sufentanil and is consequently not included in Table 1 among morphine use and we considered the unit as none user of morphine.

Raw data for IT and EDA opioid survey Click here for additional data file. Copyright: © 2017 Hein A et al. 2017 Data associated with the article are available under the terms of the Creative Commons Zero "No rights reserved" data waiver (CC0 1.0 Public domain dedication).

## Discussion

We found that all responding units used opioid supplementation and two thirds of responding units obstetric anaesthesia units in Sweden used spinal/epidural morphine as adjunct to local anaesthesia for perioperative care of CS and hysterectomies. Spinal use was rather uniform with 100 µg for CS and up to 200 µg for hysterectomies of intrathecal morphine combined with a low dose of fentanyl or sufentanil. Epidural morphine was used to a lesser extent. Opioids, such as sufentanil were also commonly added to labour analgesia, but no unit used morphine as an adjunct in labour analgesia. The common reason for withholding spinal/epidural opioid was the risk for respiratory depression, and thus the demands for post procedural monitoring.

There were some obvious differences in practice in the different units. Winther *et al.* found likewise inconsistencies in clinical guidelines for obstetric anaesthesia for CS ^7^. The most effective method for providing pain relief during labour is epidural analgesia ^8^. There are good opportunities to get a labour epidural in Sweden for every woman who wish for one since it was legislated 1971. In countries with limited resources, a single shot spinal anaesthesia may be a feasible option. Combination of low dose morphine, fentanyl and bupivacaine or morphine, sufentanil and bupivacaine are suggested to achieve effective analgesia with a prolonged effect ^9^. However, we were previously unable to find a major advantage of the addition of morphine, when comparing 0, 50 or 100 μg morphine added to 1.25 mg bupivacaine and 5 μg sufentanil during established labour, as this did not show a significant increased duration of analgesia ^10^. Morphine is not a preferred drug for labour epidural analgesia and questions about the addition of opioids other than morphine to labour epidurals were not included in this survey. Adding sufentanil or fentanyl is common practice, and may be seen more or less as the gold standard ^11, 12^. Sufentanil is the most common opioid added in labour anaesthesia in Sweden, which was reported in a national obstetric anaesthesia meeting from a survey in 2009 (Data on file).

Spinal anaesthesia is the preferred analgesia in CS when a labour epidural is not in place to top-up for perioperative use. Long acting spinal opioids as a component of the CS spinal anaesthesia has been proven superior as the post-caesarean analgesia over systemic counterparts, and made them a commonly used part of multimodal analgesic regimes ^13^. In Sweden, the long acting opioid most commonly used in CS is morphine, and we found that 63% of units used intrathecal morphine as routine in CS spinal analgesia. Since larger units with higher numbers of CS more commonly used intrathecal morphine, the impact of the routine was even more pronounced. The units that used intrathecal morphine as routine adjunct to local anaesthesia in CS, covered an estimated 73% of all CS annually performed in responding units. All responding units used intrathecal morphine doses of 100–125 µg. Palmer *et al.* found in a dose response study that morphine at 100 µg added to hyperbaric bupivacaine (12.75 mg) provided analgesia comparable to that provided by doses as high as 500 µg ^1^. The optimal effective dose for CS is not well defined. The balance between analgesia and side effects must be taken into account. The occurrence of pruritus, has been found to be dose related ^1, 6, 14^. For nausea and vomiting the relationship is not that clear but nausea and vomiting is seen in a higher frequency in higher doses than in lower dose groups ^14^.

Intrathecal morphine is suggested to provide better pain control after CS than opioid-free epidural analgesia ^15^. Intrathecal morphine has also been shown superior regarding postoperative pain relief, as compared to abdominal wall block (TAP) ^13^. The more lipophilic diamorphine was the most commonly used opioid in a survey conducted in the UK in 2008 ^5^. Diamorphine has a more rapid onset of action than morphine because of a high lipophilicity (octanol-water coefficient =280) ^16^. Diamorphine is metabolised to morphine with long-acting activity; however, diamorphine is not registered/available as pharmaceutical drug in Sweden ^16^. Fentanyl and sufentanil are the available lipid-soluble opioids used, with a quick onset for improved perioperative spinal and epidural analgesia ^17^. A combination of one lipid-soluble opioid and the long-acting water-soluble morphine is added to hyperbaric bupivacaine, which was found to be commonly used in Sweden. It is an attractive combination providing a quick onset with improved perioperative quality and a long- acting postoperative analgesia ^16^. Yet currently, the present study found that twelve units use solely lipophilic opioid adjuncts with rapid onset and shorter duration to local anaesthesia and two of the units add morphine to local anaesthetics with no addition of fentanyl or sufentanil. Intrathecal.lipophilic opioids in CS are not as effective as intrathecal morphine, they still proved to be beneficial during the period of highest analgesic demand after CS ^18^.

When a functional labour epidural is in place, it’s recommended to convert this to a full epidural anaesthesia if an emergency CS becomes necessary ^19^. Twelve of the 32 units added morphine to the epidural local anaesthesia, the most common dose was 2 mg. Singh *et al.* found epidural morphine at 1.5 mg provided none-inferior post caesarean analgesia and caused fewer adverse effects compared with 3 mg epidural morphine ^20^. Still the majority of responding units decided not to add morphine in epidural analgesia for CS postoperative analgesia. The addition of sufentanil (15 units) or fentanyl (9 units) was more common, together covering 74% of CS performed annually in responding clinics. Malhotra *et al.* found no support for adding 75 µg fentanyl in top-up epidural for CS regarding time to onset nor quality of analgesia in women already receiving epidural fentanyl during labour ^21^. Others argue the addition of epidural opioids fastens onset and enhance postoperative analgesia after CS ^22, 23^.

The addition of morphine intrathecal or epidural as adjunct for intra as well as postoperative pain relief is also regarded by many as the ‘gold-standard’, due to its positive analgesic interaction with local anaesthetics and prolonged duration of action ^6^. Spinal anaesthesia with added morphine was used in 63% of responding units for hysterectomy perioperative analgesia. The use of intrathecal morphine analgesia, sometimes as complement to general anaesthesia, has gained popularity in Sweden due to its simplicity to administer with no need for epidural or patient control analgesia infusions, and good results regarding postoperative pain ^2^. The most common dose used was 200 µg, which is the well in line with the optimal dose found in a study of the three doses (100 µg, 200 µg and 300 µg) versus placebo in abdominal hysterectomy ^2^. Intrathecal morphine use in hysterectomy varied regarding type of surgical technique: 5 units used intrathecal morphine in abdominal, vaginal and laparoscopic hysterectomies, while 7 units used intrathecal morphine in abdominal and vaginal, but not in laparoscopic, hysterectomy.

All responding units had guidelines regarding postoperative monitoring after neuraxial opioids. The routine of dividing the postoperative monitoring between a more monitoring intensive postoperative ward for the first 2–6 hours and a regular surgical ward for the following hours, up to 12 hours, according to the guidelines of SFAI, was common, and was found to be already routine in a European survey in 1996 by Rawal *et al* ^24^. In 8 units, CS were initially monitored for 2–6 hours in the obstetrical ward. Two units using intrathecal morphine for CS monitored postoperatively only in a regular maternity ward for the whole period. Seven of the 12 units that did not to use spinal/epidural morphine described a risk of respiratory depression and difficulties to monitor postoperatively as the main reason for withholding its use. In these units, which did not use morphine because of risk for respiratory depression and difficulties in postoperative monitoring, approximately 15% of CS reported were performed.

Respiratory depression is the most serious side effect associated with neuroaxial morphine; however, its occurrence is rare ^6^. In 2010, the subject “ *All patients receiving neuraxial morphine should be monitored with continuous pulse oximetry*” was debated during Controversies in Obstetric Anesthesia ^25^. It was commented that a requirement for pulse oximetry might decrease the use of neuraxial morphine as post caesarean analgesia and risk more pain in patients with more parenteral morphine consumed with the risk of increased risk of respiratory depression ^6^. We have in our institution some 15 years’ experience of approximately 2000 annual CS performed, the majority in spinal anaesthesia using a combination of fentanyl (10 µg), morphine (100 µg) and hyperbaric bupivacaine (10–12 mg). Our routine is an initial 2–3 postoperative hours monitoring in a postoperative ward, and the following hours up to 12 hours in the maternity regular ward with midwife hourly supervision of level of consciousness and in case of sedated (sleeping) patient monitoring of respiratory rate, according to the guidelines of SFAI. In the case of an unstable patient, morbid obesity or known sleep apnoea, the patient stays in the postoperative department for a longer time, determined on an individual basis. In line with responding units in this survey, we have no known experience from severe respiratory depression.

The return rate of the questionnaire was 32/47 units (68%), a figure in line with previous questionnaire survey around anaesthesia practice in conjunction to obstetrics ^26, 27^. Considering the size of the different units, the response routines cover approximately 82% out of 111,364 annual deliveries, as seen in The Swedish Medical Birth Register (2013), and 83% of CS are performed in responding units.

## Limitations

 Our main interest was to find the routine use of morphine in CS, since we learned from anaesthesia meetings with colleges that this routine, regarded as “gold standard” was not in use in several units, due to fear of respiratory depression ^6^. The questionnaire was answered by anaesthesiologists in charge of obstetric anaesthesia routines in the departments but the presented routines may not show the whole truth about general choice of treatment since implementation of routines vary. We included questions regarding spinal/epidural addition of fentanyl and sufentanil as well, but did not in detail ask for monitoring after these adjuvants when morphine was not included. We postulated postoperative monitoring was practiced according to the guidelines of SFAI and defined in brackets regarding intrathecal morphine “pain by visual analogue scale, sedation, and in case of sedation respiratory rate monitoring for 12 hours”. Our questions about monitoring focused mainly on time and location and we did not ask for more information about how the monitoring was specified and conducted. We asked for numbers of performed CS and hysterectomies, including morphine, for each unit. However, those numbers were not possible to extract from the questionnaire because of few answers. When the majority of anaesthesia units are reporting anaesthesia to the Swedish PeriOperative Register ( SPOR), hopefully in the coming years there will be better opportunities to answer questions about common routines and outcomes. We included one question regarding knowledge of serious complications in the unit associated with neuraxial anaesthesia. The quality of the obtained negative answers from this question may be considered low but we were interested in known complications. If serious complications had occurred, it is likely that the anaesthesiologist in charge would know.

## Conclusion

The use of neuraxial opioids is widely spread in Sweden; however somewhat varying regimes exists, where some units chose to use only lipophilic opioids with a rapid onset and few use only long-acting water-soluble morphine. However, a majority use a combination together with local anaesthesia. Still in some units the use of morphine is restricted because of the concern for respiratory depression and difficulties in monitoring.

## Data availability

The data referenced by this article are under copyright with the following copyright statement: Copyright: © 2017 Hein A et al.

Data associated with the article are available under the terms of the Creative Commons Zero "No rights reserved" data waiver (CC0 1.0 Public domain dedication). 

Dataset 1: Raw data for IT and EDA opioid survey. IT, intrathecal; EDA, epidural; MO, morphine. doi, 10.5256/f1000research.10705.d151418 ^28^

## Supplementary material

Supplementary File 1: Questionnaire in Swedish.

.

Supplementary File 2: Questionnaire in English.
