# Supplementary material for: Neuraxial opioids as analgesia in labour, caesarean section and hysterectomy: A questionnaire survey in Sweden
Source: F1000Res. 2017 Mar 28;6:133. Originally published 2017 Feb 13. [Version 2] doi: 10.12688/f1000research.10705.2 (PMC5381617; doi:10.12688/f1000research.10705.2)
Supplement: Supplementary file 2 [file f1000research-6-12146-s0001.tgz › a4fdce07-6bdc-495b-90c5-74ed2712abb1.docx]

**Enkät morfin intratekalt / epiduralt inom kvinnokliniken**

*Syftet med denna enkät undersökning är att kartlägga användandet av morfin intratekalt och epiduralt i samband med obstetriska och gynekologiska operationer i Sverige och förekomsten av kända incidenter av andningsdepression kopplat till detta.*

- V.g. ange det alternativ som bäst stämmer överens. Om du svarar i **word fil** skriv RÖD text, markera önskat/-de alternativ RÖTT, spara och maila bifogad word fil med svar.
- Alternativt skriv ut **pdf fil** och skriv svar, scanna och maila eller posta utskriven ifylld fil.

Svaren kommer att hanteras konfidentiellt och sjukhusets identitet används för att registrera att svar inkommit.

***Tack för din medverkan.***

|  | Frågor | Svar | |
| --- | --- | --- | --- |
| 1 | Jag arbetar på sjukhus: | _______________________________________________________________________________________ | |
| 2 | Använder ni intratekalt / epiduralt morfin vid obstetriska och/ eller gynekologiska operationer?  (flera alternativ möjliga) | 1. Ja vi använder it morfin vid obstetriska operationer 2. Ja vi använder it morfin vid gynekologiska operationer 3. Ja vi använder epiduralt morfin vid obstetriska operationer 4. Ja vi använder epiduralt morfin vid gynekologiska operationer 5. Nej vi använder inte it/epiduralt morfin vid obstetriska och/eller gynekologiska operationer | |
| 3 | Har ni skrivna riktlinjer avseende morfin intratekalt SPA/ epiduralt EDA vid obstetriska och eller gynekologiska operationer? | 1. Ja, vi har skriftliga riktlinjer (pm)avseende   intratekalt morfin vid obstetriska op   1. Ja, vi har skriftliga riktlinjer (pm) avseende epiduralt morfin vid obstetriska op 2. Ja, vi har skriftliga riktlinjer (pm)avseende   Intratekalt morfin vid gynekologiska op   1. Ja, vi har skriftliga riktlinjer (pm) avseende epiduralt morfin vid gynekologiska op 2. Nej vi saknar skriftliga riktlinjer (pm) avseende morfin intratekalt / epiduralt inom obstetriska och/ eller gyn. op. | |
| 4 | Om svar Nej ovan: Varför använder ni inte intratekalt SPA/epiduralt EDA morfin vid er enhet till obstetriska och/eller gynekologiska operationer?  (Fyll i det tillämpliga svar som stämmer bäst vid din enhet) | 1. Vi är nöjda med vår nuvarande postoperativa smärtlindring 2. Risk för andningsdepression. Vi har inte möjlighet att arrangera godtagbar postoperativ övervakning enligt SFAIs riktlinjer*(sedering, andningsfrekvens vid sedation, smärtintensitet 12 timmar) av patienterna. 3. Risk för övriga biverkningar överväger nyttan. 4. Annat: ___________________________________ | |
| 5 | Hur många patienter fick intratekalt morfin totalt under 2013 på din enhet? | C:a _______(ungefärligt antal) patienter fick intratekalt morfin | |
| *[www.sfai.se](http://www.sfai.se) - Riktlinjer – Medicinska råd och riktlinjer - Anestesi – Postoperativ smärtlindring s 7 | | | |
| 6 | Hur många patienter fick epiduralt morfin totalt under 2013 på din enhet? | C:a _______(ungefärligt antal) patienter fick epiduralt morfin | |
| **SECTIO i spinal bedövning** | | | |
| 7 | Använder ni på din enhet morfin intratekalt till sectio i spinalbedövning ? | 1. Ja morfin it till alla sectio i spinalbedövning 2. Ja morfin it till alla (elektiva och akuta)sectio i spinalbedövning där det inte föreligger asfyxirisk för barnet 3. Ja morfin till elektiva sectio i spinalbedövning (ej till akuta) 4. Annat : ___________________________________________________________________________________________________ 5. Nej | |
| 8 | Hur många sectio fick morfin intratekalt 2013 vid din enhet c:a ? | C:a _______(antal) sectio fick morfin intratekalt 2013 | |
| 9 | Vilken dos intratekalt morfin använder ni som rutin till sectio i spinalbedövning? | 1. 75 μg 2. 100 μg 3. 125 μg | 1. 150 μg 2. 200 μg 3. Annat, dos :___________μg |
| 10 | Använder ni intratekalt sufentanil/fentanyl som rutin till sectio spinalbedövning? | 1. Ja, sufentanil ges intratekalt som rutin till sectio i spinalbedövning, dos :_________μg 2. Ja, fentanyl ges intratekalt som rutin till sectio i spinalbedövning, dos: _________μg 3. Nej | |
| **SECTIO i epidural bedövning** | | | |
| 11 | Använder ni på din enhet morfin epiduralt som rutin till sectio som genomförs i epiduralbedövning? | 1. Ja morfin epiduralt till alla sectio som genomförs i epiduralbedövning, dos :_________μg 2. Ja, morfin epiduralt till alla sectio i epiduralbedövning där det inte är asfyxi risk för barnet, dos :_________μg 3. Annat:_______________________________________________________________________________________________________________________________________________ | |
| 12 | Hur många sectio fick morfin epiduralt 2013 vid din enhet? | C:a _______(antal) sectio fick morfin epiduralt 2013 | |
| 13 | Vilken dos epiduralt morfin använder ni som rutin till sectio i epiduralbedövning? | 1. 1 mg 2. 1,5 mg 3. 2 mg 4. 2,5 mg | 1. 3 mg 2. 3,5 mg 3. 4 mg 4. Annat: dos ___________μg |
| 14 | Använder ni epiduralt sufentanil/fentanyl som rutin till sectio epiduralbedövning? | 1. Ja, sufentanil ges epiduralt som rutin till sectio i epiduralbedövning, dos _________μg 2. Ja, fentanyl ges epiduralt som rutin till sectio i epiduralbedövning, dos _________μg 3. Nej | |
| **FÖRLOSSNING Spinal som smärtlindring** | | | |
| 15 | Använder ni på din enhet morfin intratekalt i spinal som smärtlindring vid förlossning**?** | 1. Ja, morfin ges i förlossningsspinal, dos ________μg 2. Nej | |
| 16 | Hur många förlossnings spinaler fick morfin intratekalt 2013 vid din enhet c:a ? | C:a _______(antal) förlossningsspinaler fick morfin intratekalt 2013 | |
| **HYSTERECTOMI** | | | |
| 17 | Hur många hysterectomier utfördes 2013 på din enhet? | C:a _______(antal) hysterectomier utfördes 2013 | |
| 18 | Använder ni på din enhet morfin intratekalt SPA/epiduralt EDA vid hysterectomi?  (Fyll i alla tillämpliga svar som stämmer vid din enhet) | 1. Ja SPAmorfin rutin till alla abdominella hysterectomier 2. Ja SPAmorfin rutin till alla vaginala hysterectomier 3. Ja SPAmorfin rutin till alla laparoskopiska hysterectomier 4. Ja EDAmorfin rutin till alla abdominella hysterectomier 5. Ja EDA morfin rutin till alla vaginala hysterectomier 6. Ja EDA morfin rutin till alla laparoskopiska hysterectomier 7. Annat : ______________________________________________________________________________________________________ 8. Nej | |
| 19 | Hur många hysterectomier fick morfin intratekalt/epiduralt 2013 vid din enhet c:a ? | C:a _______(antal) hysterectomier fick morfin intratekalt 2013  C:a _______(antal) hysterectomier fick morfin epiduralt 2013 | |
| 20 | Vilken dos/doser intratekalt/epiduralt morfin använder ni som rutin till hysterectomi(SPA)?  (Fyll i alla tillämpliga svar som stämmer vid din enhet) | 1. 75 μg 2. 100 μg 3. 125 μg 4. 150 μg 5. 200 μg | 1. 250 μg 2. 300 μg 3. Annat, dos ________μg / ________mg epiduralt |
| 21 | Användes morfin intratekalt /epiduralt till annan gynekologisk kirurgi vid din enhet 2013 | 1. Ja intratekalt SPA morfin används även till _____________________________________________,   c:a __________ (antal) patienter 2013   1. Ja epiduralt EDA morfin används även till _____________________________________________   c:a __________ (antal) patienter 2013   1. Nej | |
| **POSTOPERATIV övervakning** | | | |
| 22 | Har ni lokala riktlinjer (pm) för postop övervakning efter morfin intratekalt/epiduralt | 1. Ja 2. Nej | |
| 23 | Hur övervakas patienter efter sectio som får intratekalt morfin?  *Här efterfrågas dels* ***hur många timmar*** *patienten övervakas samt* ***var*** *patienten övervakas. Ex: Patienten övervakas först 6 timmar på postop timme 0 -6 och därefter 6 timmar på BB p.op timme 6 - 12*  *[www.sfai.se](http://www.sfai.se) - Riktlinjer – Medicinska råd och riktlinjer - Anestesi – Postoperativ smärtlindring s 7 | 1. Övervakning enligt SFAIs riktlinjer* på postoperativ avdelning ______ (antal) timmar, postop timme 0 till och med timme _________. 2. Övervakning enligt SFAIs riktlinjer* på förlossningsavdelning ______ (antal) timmar, postop. timme ______till och med postop. timme ______. 3. Övervakning enligt SFAIs riktlinjer* på BB ______ (antal) timmar, postop. timme ______till och med postop. timme ______. 4. Övervakning på annat sätt ___________________________________________________ | |
| 24 | Hur övervakas patienter efter sectio som får epiduralt morfin? | 1. Övervakning enligt SFAIs riktlinjer* på postoperativ avdelning ______ (antal) timmar, postop. timme 0 till och med postop. timme ______. 2. Övervakning enligt SFAIs riktlinjer* på förlossningsavdelning _______ (antal) timmar, postop. timme ______till och med postop. timme ____. 3. Övervakning enligt SFAIs riktlinjer* på BB _____(antal) timmar, postop. timme ______till och med postop. timme ______. 4. Övervakning på annat sätt ___________________________________________________ | |
| 25 | Hur övervakas patienter efter hysterectomi/annan gynekologisk op som får intratekalt morfin? | 1. Övervakning enligt SFAIs riktlinjer* på postoperativ avdelning ______(antal) timmar, postop. timme 0 till och med postop. timme _____. 2. Övervakning enligt SFAIs riktlinjer* på vårdavdelning ____(antal) timmar, postop. timme _____till och med postop. timme ______. 3. Övervakning på annat sätt ___________________________________________________ | |
| 26 | Har det förekommit någon / några för dig känd/a incident/er avseende (postoperativ ) respiratorisk andningsdepression i kombination med intratekalt / epiduralt morfin vid din enhet ? (använd ökat utrymme vb) | 1. Ja 2. Nej   Om Ja, beskriv, antal tillfällen _________________ beskriv incident/er ___________________________________ ___________________________________________________________ ____________________________________________________________________________________________________________________________________________________________________________________________________________________________________________ | |

***Vi tackar för din medverkan och är tacksamma för ditt inskickade svar!***

*Anette Hein, överläkare KI DS - 08 12357501*

*hein.enkatsvar@gmail.com*

*Anestesi och IVA Danderyds sjukhus*

*182 88 Stockholm*

[*anette.hein@ds.se*](mailto:anette.hein@ds.se)

*Jan Jakobsson, adj. professor, KI DS*

*Caroline Haegerstrand docent KI DS,*

*Gunnar Dahlgren med dr, KI, S:t Göran sjh*
