# Supplementary material for: Neuraxial opioids as analgesia in labour, caesarean section and hysterectomy: A questionnaire survey in Sweden
Source: F1000Res. 2017 Mar 28;6:133. Originally published 2017 Feb 13. [Version 2] doi: 10.12688/f1000research.10705.2 (PMC5381617; doi:10.12688/f1000research.10705.2)
Supplement: Supplementary file 3 [file f1000research-6-12146-s0002.tgz › b1d4416f-df35-44ca-ae31-c75bcf1dda31.docx]

**Survey intrathecal / epidural morphine in labour and postoperative after Caesarean section and hysterectomy**

*The aim with this survey is to assess the use of intrathecal and epidural opioids in obstetric and gynaecological patients in Sweden and known incidents from severe respiratory depression linked to neuroaxial opioids.*

- Please mark the alternative with best match to your routine.
- If you respond in a **word file** write RED text, mark the chosen alternative /-s RED, save and mail attatched word file including your answers.
- Or print the **pdf file** write your answers, scan and mail or post.

The returned answers will be handled confidentially. The hospitals identity will be used only to register returned answers.

***Thank you for participation.***

|  | Questions | Answers | |
| --- | --- | --- | --- |
| 1 | I work in hospital: | _______________________________________________________________________________________ | |
| 2 | Do you use intrathecal / epidural morphine in obstetrical and / or gynaecological operations?  (more than one answer is posible) | 1. Yes we use it morphine in obstetrical operations. 2. Yes we use it morphine in gynaecological operations. 3. Yes we use epidural morphine in obstetrical operations. 4. Yes we use epidural morphine in gynaecological operations. 5. No we don’t use it / epidural morphine in obstetrical and/ or gynaecological operations. | |
| 3 | Have you written guidelines regarding morphine intratecal SPA/ epidurally EDA in obstetrical and/ or gynaecological operations.? | 1. Yes we have written guidelines regarding intrathecal morphine in obstetrics 2. Yes we have written guidelines regarding epidural morphine in obstetrics 3. Yes we have written guidelines regarding intrathecal morphine in gynaecological operations. 4. Yes we have written guidelines regarding epidural morphine in gynaecological operations. 5. No we don’t have guidelines regarding it / epidural morphine in obstetrical and/ or gynaecological operations. | |
| 4 | If answer No in question no 3 Why don’t you use it/ EDA morphine in your unit in obstetrical and/ or gynaecological operations.?  (Please chose the answer with best match in your unit) | 1. We are satisfied with our postopertive analgesia 2. Risk for respiratory depression. We don’t have the opportunities to arrange postoperative monitoring in accordance with national guidelines (sedation, respiratory frequency if sedated, painintensity 12 hours) 3. We find the risk for other side effects outweighs the favor 4. Other: ___________________________________ | |
| 5 | How many patients got intrathecal morphine during 2013 in your unit? | _______(about) number of patients got intrathecal morphine during 2013 | |
| *[www.sfai.se](http://www.sfai.se) - Guidelines – ”Medical advice and guideline - Anaethesia– Postoperative pain p 7” | | | |
| 6 | How many patients got epidural morphine during 2013 in your unit? | _______(about) number of patients got epidural morphine during 2013 | |
| **Ceaseran SECTIO i spinal anaesthesia** | | | |
| 7 | Do you use intrathecal morphine to cesarean section spinal anaesthesia in your unit routinly? | 1. Yes, it morphine to all CS in spinal anaesthesia 2. Yes, it morphine to all CS in spinal anaesthesia (elective and emergency) when there is no risk of asphyxia for the baby 3. Yes, it morphine to all elective CS in spinal anaesthesia (but not to emergency CS) 4. Other: ___________________________________________________________________________________________________ 5. No | |
| 8 | How many Cesarean Section got intrathecal morphine during 2013 in your unit? | About _______(number) cesarean section got intrathecal morphine during 2013 | |
| 9 | What dose of intrathecal morphine do you routinly use for cesarean section spinal anaesthesia? | 1. 75 μg 2. 100 μg 3. 125 μg | 1. 150 μg 2. 200 μg 3. Other, dose:_________μg |
| 10 | Do you routinly use intrahtecal sufentanil/fentanyl for cesarean section spinal anaesthesia? | 1. Yes sufentanil i t is used as routine for cesarean section spinal anaesthesia, dose :_________μg 2. Yes fentanyl i t is used as routine for cesarean section spinal anaesthesia, dose :_________μg 3. No | |
| **Ceaseran SECTIO in epidural anaesthesia** | | | |
| 11 | Do you use epidural morphine to cesarean section performed in epidural anaesthesia in your unit routinly? | 1. Yes epidural morphine is used as routine for all cesarean section performed in epidural anaesthesia, dose :_________μg 2. Yes epidural morphine is used as routine for all cesarean section performed in epidural anaesthesia, when there is no risk of asphyxia for the baby dose :_________μg 3. Other:_   ____________________________________________________________________________________________________   1. No__________________________________________ | |
| 12 | How many Cesarean Section got intrathecal morphine during 2013 in your unit? | About _______(number) cesarean section got intrathecal morphine during 2013 | |
| 13 | What dose of epidural morphine do you routinly use for cesarean section epidural anaesthesia? | 1. 1 mg 2. 1.5 mg 3. 2 mg 4. 2.5 mg | 1. 3 mg 2. 3.5 mg 3. 4 mg 4. Other: dose ___________μg |
| 14 | Do you routinly use epidural sufentanil/fentanyl for cesarean section epidural anaesthesia? | 1. Yes sufentanil epidurally is used as routine for cesarean section epidural anaesthesia, dose :_________μg 2. Yes fentanyl epidurally is used as routine for cesarean section epidural anaesthesia, dose :_________μg 3. No | |
| **Vaginal birth Spinal as analgesia during delivery** | | | |
| 15 | Do you use intrathecal morphine as spinal analgesia during vaginal birth in your unit routinly? | 1. Yes, it morphine is used in spinal analgseia 2. dose ________μg 3. No | |
| 16 | How many patients got spinal analgesia including morphine during vaginal birth in your unit 2013? | About _______(number) patients got spinal analgesia including morphine during vaginal birth in 2013 | |
| **HYSTERECTOMY** | | | |
| 17 | How many hysterectomies were performed 2013 in your unit? | About _______(number) hysterectomies were performed 2013 | |
| 18 | Do you use morphine intrathecally, SPA /epidurally, EDA in hysterectomies in your unit?  (Mark all applicable answers ) | 1. Yes intrathecal morphine is used routinely to all abdominal hysterectomies 2. Yes intrathecal morphine is used routinely to all vaginal hysterectomies 3. Yes intrathecal morphine is used routinely to all laparoscopic hysterectomies 4. Yes epidural morphine is used routinely to all abdominal hysterectomies 5. Yes epidural morphine is used routinely to all vaginal hysterectomies 6. Yes epidural morphine is used routinely to all laparoscopic hysterectomies 7. Other: ______________________________________________________________________________________________________ 8. No | |
| 19 | How many hysterectomies got morphine intrathecally /epidurally in your unit 2013? | About _______(number) hysterectomies got morphine intrathecally 2013  About _______(number) hysterectomies got morphine epidurally 2013 | |
| 20 | What dose of intrathecal/epidural morphine do you routinly use for hysterectomy in your unit?  (Mark all applicable answers ) | 1. 75 μg 2. 100 μg 3. 125 μg 4. 150 μg 5. 200 μg | 1. 250 μg 2. 300 μg 3. Annat, dos ________μg / ________mg epiduralt |
| 21 | Was intrathecal/epidural morphine used in other kind of gynaecological surgery in your unit 2013 ? | 1. Yes intrathecal morphine was also used in _____________________________________________,   About __________ (number) patients 2013   1. Yes epidural morphine was also used in _____________________________________________,   About __________ (number) patients 2013   1. No | |
| **POSTOPERATIVE surveillance** | | | |
| 22 | Do you have local guidelines regarding postoperative surveillance after intrathecal/ epidural morphine? | 1. Yes 2. No | |
| 23 | How is patient surveillance organised after cesarean section in spinal anesthesia with intrathecal morphine?  *Here we ask for* ***how many hours*** *the patient is monitored and* ***were*** *the patient is localised.*  *E.g. Patient is first monitored 6 hours in postoperative department for postoperative hour 0 – 6 and after that 6 h in regular ward during hour 6-12*  *[www.sfai.se](http://www.sfai.se) – ”Riktlinjer – Medicinska råd och riktlinjer - Anestesi – Postoperativ smärtlindring” p 7 | 1. Surveillance by SFAI guidelines* in postoperative ward ____ (number) hours, during postoperative hour 0 to _______ (h) 2. Surveillance by SFAI guidelines* in maternety ward ____ (number) hours,   during postoperative hour ______ to_______ (h)   1. Surveillance by SFAI guidelines* in regular ward ____ (number) hours,   during postoperative hour ______ to_______ (h)   1. Other: ___________________________________________________ | |
| 24 | How is patient surveillance organised after cesarean section in epidural anesthesia with epidural morphine? | 1. Surveillance by SFAI guidelines* in postoperative ward ____ (number) hours, during postoperative hour 0 to _______ (h) 2. Surveillance by SFAI guidelines* in maternety ward ____ (number) hours,   during postoperative hour ______ to_______ (h)   1. Surveillance by SFAI guidelines* in regular ward ____ (number) hours,   during postoperative hour ______ to_______ (h)   1. Other: ___________________________________________________ | |
| 25 | How is patient surveillance organised after hysterectomy / other gynecological operation where intrathecal morphine has been administered? | 1. Surveillance by SFAI guidelines* in postoperative ward ____ (number) hours, during postoperative hour 0 to _______ (h) 2. Surveillance by SFAI guidelines* in regular ward ____ (number) hours,   during postoperative hour ______ to_______ (h)   1. Other: ___________________________________________________ | |
| 26 | Do you know any incidents regarding postoperative respiratory depression in combination with intrathecal / epidural morphine in your department? | 1. Yes 2. No   If yes, please describe number of incidents__________ and what happended _________________________________ ___________________________________________________________ ___________________________________________________________ ____________________________________________________________________________________________________________________________________________________________________________________________________________________________________________ | |

***Thank you for participation!***

*Anette Hein, MD consultant*

*KI DS 046(0)8 12357501*

*hein.enkatsvar@gmail.com*

*Anaesthesia and intensive care Danderyd hospital*

*182 88 Stockholm*

*anette.hein@sll.se*

*Jan Jakobsson, MD, adj. professor, KI DS*
