# Supplementary material for: Neuraxial opioids as analgesia in labour, caesarean section and hysterectomy: A questionnaire survey in Sweden
Source: F1000Res. 2017 Mar 28;6:133. Originally published 2017 Feb 13. [Version 2] doi: 10.12688/f1000research.10705.2 (PMC5381617; doi:10.12688/f1000research.10705.2)
Supplement: Peer review history

# Peer review: Referee response for version 2

**DOI:** 10.5256/f1000research.12146.r21306

**Published:** 2017-03-30

**Authors:** Wojciech Weigl

**Affiliations:** 1 Department of Surgical Sciences/Anaesthesiology and Intensive Care, Uppsala University, Uppsala, Sweden

Thank you for correcting the manuscript. In my opinion, the manuscript has improved significantly and all comments have been addressed. The aim of the study and the results are presented in a clear manner. Table 1 has improved greatly!

I have read this submission. I believe that I have an appropriate level of expertise to confirm that it is of an acceptable scientific standard.

# Peer review: Referee response for version 1

**DOI:** 10.5256/f1000research.11542.r20178

**Published:** 2017-03-08

**Authors:** Wojciech Weigl

**Affiliations:** 1 Department of Surgical Sciences/Anaesthesiology and Intensive Care, Uppsala University, Uppsala, Sweden

The study of Hein et al. presents results of a nation-wide survey on the use of neuraxial opioids in obstetric and gynecology patients in Sweden. I found especially interesting these parts that were devoted to cesarean section (CS). Multimodal analgesia became a widely used approach to post-CS pain management and the use of intrathecal opioids is one of the important components in this approach. Among intrathecal opioids, low-dose morphine appear to be the most widely recommended ^1^. However, this hydrophilic opioid has a high frequency of adverse effects ^2^ such us nausea and vomiting, pruritus, and potentially serious late respiratory depression ^3^. That’s why the question of the current routines related to this topic in Sweden is important in daily life clinical practice.

 I think that several things could be corrected to improve quality of the paper.

General comments:

 Authors should choose tense in which they present and discuss findings. Personally, I don’t like to present tense because, as stated below it can be sometimes misleading. Anyway, consistency is needed.

 English language could also be improved. There are some awkward expressions such as ’fast opioid’ which is not really medical term.

Specific comments:

 Title:

 The title could be more clear and in better English.

 Abstract:

 I recommend to maintain consistency regarding the aim of the study, whether it is the use of neuraxial opioids or just morphine.

 ‘the patient is in postoperative care or an obstetrical ward over 2-6 hours and up-to 12 hours in an ordinary surgical ward.’ I would add ‘is monitored in …’

 Introduction

 ‘Intrathecal and epidural morphine improve postoperative analgesia after caesarean section (CS) and hysterectomy and intrathecal labour analgesia’. Did the authors mean during intrathecal labour analgesia? This is at least what Yeh et al. had in mind during their study.

 Methods:

 ‘A questionnaire survey was sent to anaesthesiologists in charge of Swedish obstetric anaesthesia’ I think it sounds a bit strange. Is it not more simple just to write that the questionnaire was sent to the anaesthesiologists in charge of obstetric anaesthesia units in hospitals in Sweden? Furthermore, information about anesthesiologist is repeated 3 times.

 What is ‘Swedish Association of Obstetric Anaesthesia and Intensive Care’?

 The questionnaire (Supplementary File 1 and Supplementary File 2) was produced to reflect how common the use of intrathecal and epidural morphine, fentanyl and sufentanil as adjunct to local anaesthetics for perioperative care of CS and hysterectomy is, and also the routine use of intrathecal morphine for labour analgesia. I think this sentence could be corrected in underlined parts as it sounds awkward.

 ‘specify the numbers of patients administered with opioids, including neuraxial morphine, for the operations performed’ it is not clear if the authors were interested in intrathecal morphine alone or in other intrathecal opioids or, what would be even more interesting what was the combinations of used opioids. It is the same issue what with the aim of the study.

 Results

 ‘Epidural sufentanil. In total, 15 units use sufentanil as the fast opioid in the epidural anaesthesia for CS anaesthesia’ maybe opioid with rapid onset of action.

 50mcg of epidural sufentanil is quite a large dose. I think it should be commented.

 It is quite surprising that for hysterectomy and gynecological abdominal surgery opioids are used intrathecally and not with the use of epidural anesthesia. Can the authors comment on that?

 ‘Postoperative monitoring is generally organized within the initial 2–6 hours in the postoperative ward and the following hours, up to 12 hours, in the regular surgical ward, according to the guidelines of SFAI – Swedish Association of Anaesthesia and Intensive Care.’ Did you mean this is general routine in Sweden or this was the result of survey? Using present tense in result section is a bit misleading. Also ‘in the regular surgical ward, according to the guidelines of SFAI – Swedish Association of Anaesthesia and Intensive Care.’ I would change into: … according to the guidelines of Swedish Association of Anaesthesia and Intensive Care (SFAI).

 Postoperative monitoring: The authors write about 9 units of 32 in case of CS, what happened to patients in the rest of 20 units where morphine was used?

 ‘Seven of the eleven units that chose not to use spinal/epidural morphine’ Or was it 12/32 units which did not use morphine as in table 1.

 Discussion

 I think authors should avoid expressions such as ‘Swedish obstetric anaesthesia’ because such thing do not exist. Use instead obstetric anaesthesia units in Sweden.

 ‘Opioids were also commonly added to labour analgesia as sufentanil,’ please change for clarity into: ‘Opioids such as sufentanil were also commonly added to labour analgesia… ‘

 ‘there are good opportunities to get a beneficial epidural in Sweden’ what did you mean by ‘beneficial’? The citation should be placed earlier, as Cochrane’s review does not say much how these issues occur in Sweden.

 ‘Sufentanil is the most common opioid added in labour anaesthesia in Sweden, which was reported in a national obstetric anaesthesia meeting from a survey in 2009 (not published).’ Oral presentations that were not published should not be mentioned or cited in a good quality paper.

 ‘Spinal anaesthesia is the preferred analgesia in CS when a working epidural is not in place for conversion and top-up.’ What do you mean ‘conversion’?

 ‘Yet currently, the present study found that twelve units use solely fast opioid adjuncts to local anaesthesia and two of the units add morphine to local anaesthetics with no addition of fentanyl or sufentanil, but one of the units stated they plan to start adding fentanyl shortly.’ Last part of the sentence could be omitted; the authors should filter not important information. On the other hand it could be discussed that event though intrathecal lipophilic opioids in CS are not as effective as intrathecal morphine, they still proved to be beneficial during the period of highest analgesic demand after cesarean section ^4^.

 ‘Yet, the addition of epidural opioids has been proven to enhance postoperative analgesia after CS with earlier onset’ I don’t understand this sentence.

 Withdrawing from the use of intrathecal morphine could be discussed in context of other large studies that report usage of intrathecal opioids in obstetrics ^5^^-^^7^.

 Limitation

 Usually, at the end of manuscript, authors should state limitations of the study. Some aspects that could be discussed are:

 The postoperative monitoring routines are limited to time perspective. There is nothing about what was actually monitored and how often.

 Quality of information obtained from the question regarding complications is very low.

I have read this submission. I believe that I have an appropriate level of expertise to confirm that it is of an acceptable scientific standard, however I have significant reservations, as outlined above.

## References

[1] GomaHFlores-CarrilloJWhizar-LugoV: Spinal Additives in Subarachnoid Anaesthesia for Cesarean Section.2014; 10.5772/58851 10.5772/58851

[2] DahlJBJeppesenISJørgensenHWetterslevJMøinicheS: Intraoperative and postoperative analgesic efficacy and adverse effects of intrathecal opioids in patients undergoing cesarean section with spinal anesthesia: a qualitative and quantitative systematic review of randomized controlled trials. *Anesthesiology*.1999;91(6) :1919-27 1059863510.1097/00000542-199912000-00045

[3] AbouleishERawalNRashadMN: The addition of 0.2 mg subarachnoid morphine to hyperbaric bupivacaine for cesarean delivery: a prospective study of 856 cases. *Reg Anesth*.16(3) :137-40 1883770

[4] WeiglWBieryloAWielgusMKrzemień-WiczyńskaSSzymusikIKolaczMDabrowskiMJ: Analgesic efficacy of intrathecal fentanyl during the period of highest analgesic demand after cesarean section: A randomized controlled study. *Medicine (Baltimore)*.2016;95(24) : 10.1097/MD.0000000000003827 e3827 10.1097/MD.0000000000003827 27310958PMC4998444

[5] Orbach-ZingerSIoscovichAAviramABabytzSFeinSReuveniAEidelmanLA: National survey of postoperative pain control after cesarean delivery. *Isr Med Assoc J*.2014;16(3) :153-6 24761702

[6] JacquesVVialFLerintiuMThillyNMc NelisURaftJBouazizH: [Enhanced recovery following uncomplicated elective caesarean section in France: a survey of national practice]. *Ann Fr Anesth Reanim*.2013;32(3) : 10.1016/j.annfar.2013.01.016 142-8 10.1016/j.annfar.2013.01.016 23433933

[7] MarcusHEBehrendASchierRDagtekinOTeschendorfPBöttigerBWSpöhrF: [Anesthesiological management of Caesarean sections : nationwide survey in Germany]. *Anaesthesist*.2011;60(10) : 10.1007/s00101-011-1931-y 916-28 10.1007/s00101-011-1931-y 21833754

# Peer review: Referee response for version 1

**DOI:** 10.5256/f1000research.11542.r20179

**Published:** 2017-02-27

**Authors:** Jakob Walldén, Johan Styrke

**Affiliations:** 1 Anaesthesia and Intensive Care Section, Umeå University, Umeå, Sweden; 2 Department of Surgical and Perioperative Sciences, Umeå University, Umeå, Sweden

The present study explores the use of opioids in obstetric and gynaecological surgery in Sweden. The authors have done a nice survey among Swedish hospitals covering a majority of the procedures performed. The study is properly designed, the results reported correctly with adequate clinically relevant conclusions. However, some revisions are needed in the manuscript.

**Throughout the manuscript please revise the language according to tenses and report the results in “past time”.** Please use a consequent numbering format with numbers and letters.

 Consider sending the manuscript for language proof reading to improve the quality.

**Abstract:**

 Change “…47 anaesthesiologists at ob. units…” to “… all anaesthetic obstetric unit..”

**Introduction:**

 Line 5: 90-150 of how many??

**Methods:**

*“..26 questions sent by mail…”*  Please specify postal mail

*“…questionnaire was produced to reflect….”*  Designed or Formulated might be better than Produced

 “…no formal statistical tests WERE used.” Please use past form.

**Results**

 Regarding the responding clinics, was it 32 units that responded with postal mail? Or total response rate? Consider deleting the word “mailed”.  The results here are not in accordance with the results in the abstract where you state that the units represented 83% of annual CS.

 Better formulation may be:  “… 32 of 47 (68%) units responded to the questionnaires representing 83 % of annual CS in Sweden...”

 Please provide proof that larger units were more willing to respond. Significant? 

 Please be consequent with the dosing: now both µg and microgram are used.

 Section epidural morphine:

 Table 1: The table is difficult to view in the current layout.

 Suggestion

 -------

                         Number of units

**Response rate 32/47**

**Intrathecal morphine  20**

 Standard dose 100 µg           17

 Standard dose 125 µg            3

 Combined with fentanyl        16

 Combined with sufentanil       2

**Intrathecal Fentanyl   21**

 Standard dose 10-14 µg        18

 Etc…

 -------------

**Discussion**

 Please add “…all responding units used….” to the first sentence.

 “…a survey in 2009 (not published)”.  I “Data on file” might be a better formulation than “not published”

 “The addition of sufentanil (15 units) or fentanyl (9units…)” Please reformulate so that it is clear that you mean number of OB/Gyn units and not units of the drugs.

 Last paragraph of the Discussion: Move the results to the first part of the results section.

 Please also include limitations of the study in the discussion. Were there any pit-falls? Can the results be generalised outside Sweden? The standard care for the units are reported, but is there a variability at the units that has to be discussed?

 Consider reducing the length of the discussion.

**References**

 There is a mixed format of the references with special attention to the page numbering. Please adhere to the Index Medicus/Medline format.

 Check page number ref 23.

We have read this submission. We believe that we have an appropriate level of expertise to confirm that it is of an acceptable scientific standard.
